# Discovery Mechanisms for the Sensor Web

**DOI:** 10.3390/s90402661

**Published:** 2009-04-16

**Authors:** Simon Jirka, Arne Bröring, Christoph Stasch

**Affiliations:** Westfälische Wilhelms-Universität, Institute for Geoinformatics, Weseler Straße 253, 48151 Münster, Germany; E-Mails: arneb@uni-muenster.de; staschc@uni-muenster.de

**Keywords:** Sensor Networks, Sensor Web Enablement (SWE), Sensor Discovery

## Abstract

This paper addresses the discovery of sensors within the OGC Sensor Web Enablement framework. Whereas services like the OGC Web Map Service or Web Coverage Service are already well supported through catalogue services, the field of sensor networks and the according discovery mechanisms is still a challenge. The focus within this article will be on the use of existing OGC Sensor Web components for realizing a discovery solution. After discussing the requirements for a Sensor Web discovery mechanism, an approach will be presented that was developed within the EU funded project “OSIRIS”. This solution offers mechanisms to search for sensors, exploit basic semantic relationships, harvest sensor metadata and integrate sensor discovery into already existing catalogues.

## 1. Introduction

Within the last years the utilization of sensor networks for observing nearly any phenomenon of interest has become more and more important [1]. Sensors are used for monitoring biological, chemical or meteorological phenomena in order to build expert systems in domains like wildlife tracking, precision agriculture, risk monitoring or hazard management. Indeed, the range of sensor network applications is nearly unlimited, thus, in order to be able to flexibly integrate any kind of sensor into any type of (software) system the Sensor Web Enablement (SWE) architecture [2] of the Open Geospatial Consortium (OGC) has been developed.

The OGC SWE framework, which will be introduced in Section 2, is based on the service-oriented architectures (SOA) concept. It defines the interfaces of (web) services for accessing sensor data, for controlling sensors and for alerting based on measured sensor data. Within the last years, the SWE architecture has been advanced to a solid and mature state. Now, most of the SWE standards have been adopted as official OGC standards and several practical applications relying on the SWE standards have been built. The following selection provides a good overview on SWE applications. The CSIRO Hydrological Sensor Web (http://www.csiro.au/science/SensorsAndWaterUse.html) deals with monitoring the water cycle in Tasmania using SWE concepts. Wupperverband FluGGS system (http://fluggs.wupperverband.de/pegel/mapoverview.html) is a web based application for providing hydrological information for a river system. Also in this case the underlying technology is SWE. Another project addresses oceanographic research: The OOSTethys community, http://www.oostethys.org/, works on the integration of ocean observation systems. An important means for achieving this goal is the Sensor Web Enablement framework. Finally, the German Indonesian Tsunami Early Warning System (http://www.gitews.de/) (GITEWS) uses for certain sensor data management tasks SWE components.

However, there is still one challenge left that has to be addressed within this context: the discovery of sensors and SWE services. Although, the current OGC Catalogue Service [3] provides a good basis, several open issues resulting from the specific characteristics of sensor networks need to be solved.

Thus, in Section 3 the specific requirements that have to be taken into account when building a SWE registry or catalogues will be discussed. This includes on the one hand the often highly dynamic structure of sensor networks and sensor status information, while on the other hand, the need for specific sensor metadata formats, metadata harvesting mechanisms and semantic descriptions for observed phenomena have to be taken into account.

In Section 4 a solution developed within the EU funded project “OSIRIS” will be introduced. This solution addresses the Sensor Web discovery requirements. It comprises an approach for a sensor registry, an idea for handling the semantics of the observed phenomena, a solution for describing and harvesting sensor metadata (based on existing standards) and also an idea for linking a potential SWE discovery solution to existing OGC catalogues.

Finally, Section 5 will describe the practical experiences gained during the OSIRIS project. Within this project, the presented discovery framework was used in scenarios like forest fire fighting, monitoring of air pollution, fire detection in industrial buildings and measuring water pollution. This article will conclude in Section 6 with a short summary as well as an outlook on future research challenges.

## 2. Sensor Web

This section introduces the idea of Sensor Webs. First an introduction into the OGC will be given. After this the OGC Sensor Web Enablement architecture including its information and service models will be provided. Finally, an overview on related work outside the OGC context will be given.

### 2.1. The OGC

The Open Geospatial Consortium (OGC) is an international standardisation organization consisting of more than 370 members. It comprises partners from industry as well as universities and government agencies. The overall vision of the OGC activities is the so called Geospatial Web, which can be seen as enabling the integration of the World Wide Web with spatiotemporal data along with associated functionalities. Within the Geospatial Web it is possible to publish, discover and use geodata as well as geoprocessing services in an interoperable way.

In order to achieve the aims of the Geospatial Web, the OGC members develop interoperable data models and (web) service interface standards [4]. For designing and evolving the Geospatial Web standards, the OGC relies on an open, international and participatory consensus process. To insure broad use and accessibility, all OGC standards are provided on a royalty free, non-discriminatory basis. Through close cooperation with the International Organization for Standardization (ISO) several OGC standards have also been adopted as ISO standards.

This paper will focus on the activities of the OGC SWE Working Group. The SWE Working Group deals with the development of standards allowing the integration of sensors and sensor networks into the Geospatial Web. The aim is to extend the Geospatial Web to evolve it and develop a specialized subtype called the “Sensor Web”.

### 2.2. The Sensor Web Enablement Architecture

The Sensor Web Enablement working group has specified a number of standards that define formats for sensor data and metadata as well as sensor service interfaces. Together, these standards form a framework which allows fulfilling the goals of the Sensor Web. Main functionalities of the Sensor Web are [2]:

- Discovery of sensors and sensor data.
- Description of sensors and also measurements (e.g. reliability and accuracy).
- Access to sensor parameters.
- Access to measurement data (real-time data as well as time series) based on standardized data formats.
- Tasking of sensors.
- Alerting mechanisms based on sensor measurements and certain alert criteria.

**Figure 1.**
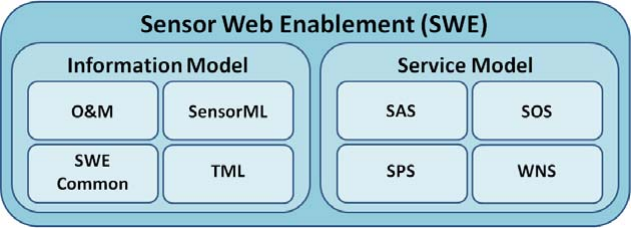
Overview of the Sensor Web Enablement architecture.

The SWE standards suite can be divided in two major subgroups: the SWE information model dealing with data formats and the service model dealing with the interfaces of (web) services. Figure 1 gives an overview of the SWE framework which will be presented in more detail within the next two subsections.

### 2.3. The SWE Information Model

Within the SWE information model, a set of standards has been defined that allows the XML based encoding of data observed by sensors as well as metadata for describing sensors. It comprises four different standards:

- SWE Common (while SWE Common is currently still a part of the SensorML standard, in the next version of SensorML, SWE Common will be a separate standards document) [5] provides means for encoding basic information building blocks that are used within the other SWE data formats.
- Observations and Measurements (O&M) [6, 7] is an encoding for data observed or measured by sensors.
- Sensor Model Language (SensorML) [5] is used for the metadata description of sensors and sensor systems (as well as abstract processes). Thus, SensorML is of special importance for building a sensor registry.
- Transducer Markup Language (TML) [8] supports the encoding of sensor data as well as metadata. However, TML is optimized for data streaming so that it addresses a different application area than O&M and SensorML. Currently TML is only rarely used and will not be taken into account for the registry solution described in this work.

With regards to sensor discovery SensorML is the most important one of the four mentioned encodings. Therefore, SensorML is introduced here in deeper detail:

The SensorML standard specifies an encoding for descriptions of sensors and sensor systems. It forms the basis for delivering the metadata needed for the discovery of sensors and sensor data. In addition, the information provided by SensorML documents can also be used for interpreting, understanding and analyzing sensor data. During the design of SensorML three main goals had to be achieved:

- For interpreting sensor data correctly, it is often necessary to know the parameters of the measurement process (e.g. information about the sensor calibration). SensorML provides a means for describing sensor parameters in order to allow the interpretation of measured data.
- In many cases sensor data is processed after it has been captured by a sensor. SensorML allows describing the processing steps that were performed on sensor data so that every user of the data can reconstruct how the data set he uses has been created.
- For supporting the discovery of sensors, SensorML provides means for encoding information about the sensor itself, its operator, its location, observed phenomena, the sensor outputs et cetera. Sensor Web services can use SensorML as an output format for describing the sensors they encapsulate. So, SensorML documents can be harvested by a sensor registry in order to populate its search indexes.

Later within this article, the use of SensorML as an input to a sensor registry will be addressed. Since, the standard is quite generic, potential use cases for SensorML are of broad range. This makes it necessary to define a profile for SensorML in order to ensure that every SensorML based sensor description contains all metadata necessary for sensor discovery.

### 2.4. The SWE Service Model

While the SWE Information Model defines encodings that are used within the SWE framework, the SWE Service Model comprises standards that specify the interfaces of the different Sensor Web services. Four interfaces of Sensor Web services have been specified:

- The Sensor Observation Service (SOS) [9] offers operations for the pull-based access to sensor measurements as well as metadata.
- The Sensor Alert Service (SAS) [10] can be used for subscribing to alerts in case of a sensor measurement event that fulfills certain user defined criteria (e.g. receiving an alert if the water level measured by a sensor rises above a specified threshold).
- The Sensor Planning Service (SPS) [11] can be used for tasking sensors and setting their measurement parameters.
- The Web Notification Service (WNS) [12] is, unlike the other three services, not directly sensor related. Instead, it is a kind of helper service which provides asynchronous notification mechanisms between SWE services and clients or other SWE services (e.g. delivering alerts to users).

All of the SWE services are based on a common OGC model for web services. Thus, they all provide the so called GetCapabilities operation for retrieving a self-description of the according service instance. The GetCapabilities operation is common to all service interfaces standardized by the OGC. It allows clients to retrieve self-descriptions of OGC compliant web service instances. This comprises for example information about the operations supported by the service instance, content that is offered by the service instance or details about the service provider. In case of SWE services this operation can for example include a list of the sensors encapsulated by the SWE service instance. Furthermore there are means (although differing among the SWE service interfaces) for retrieving SensorML based metadata descriptions which form the basis for the sensor metadata harvesting mechanisms that will be described later on.

### 2.5. Related Work

Besides the work within the OGC SWE context, several other activities regarding the topic of sensor discovery or sensor metadata management shall be mentioned. Because this paper concentrates on the OGC SWE framework, these approaches will not be directly addressed by the later presented discovery solution. In the future investigation of the integration of these approaches and a SWE discovery framework will be an interesting research topic.

An important standard that needs to be considered is the IEEE 1451 suite (http://ieee1451.nist.gov/) which provides a set of standards for describing sensors (and actuators) including their connections and their input/output signals. This set of standards provides a foundation for describing sensors on a level that is closer to the sensor hardware itself. An approach of mapping IEEE 1451 to OGC SWE standards is described in [13]. Future research has to consider how to integrate IEEE 1451 and SWE registries.

In [14] an approach for sensor metadata management is described. Within this paper, a solution is presented that relies on a wiki based platform as well as a relational database in order to manage different types of metadata. A special focus is put on describing intermediate processing steps that have been performed on a data set. This solution aims at managing and providing access to sensor metadata for ensuring a correct interpretation of sensor data.

An alternative metadata format for sensors is described in [15]. The authors propose a Sensor Description Markup Language (SDML) which can be used for describing sensors from an external perspective. Unlike SensorML, SDML does not aim at describing the internal sensor structure. Thus it can be seen as a more lightweight alternative. Furthermore in this paper an interesting approach for harvesting sensor metadata is described. It is shown how sensors that are available through web sites can be integrated into a discovery tool by crawling web sites. However, the presented approach is limited to narrow domains (it was presented in the context of traffic web cams).

Another important aspect is the discovery of sensors within peer-to-peer networks. Many sensor networks are organized using peer-to-peer models. This approach provides a high scalability compared to centralized solutions so that large sensor networks can be handled efficiently. However this creates special challenges for sensor discovery as a central search index is usually not available. To address this challenge is the aim of ongoing projects. For example in [16] a decentralized peer-to-peer GIS architecture is proposed. This architecture, which addresses GIS in general and not only sensor networks, comprises also an approach for discovering resources in peer-to-peer networks.

## 3. Requirements for Sensor Discovery

When building a sensor discovery solution several requirements have to be taken into account. First, what has to be discovered must be clearly defined: is a user interested in finding individual sensor instances or is he rather searching for SWE services which offer certain data or functionality. These two types of sensor discovery will be discussed in Section 3.1 After that, Sections 3.2 to 3.5 will discuss challenges that arise from the special characteristics of sensor networks: the dynamic structure of sensor networks, the need for taking into account sensor status information, special formats and harvesting mechanisms for sensor metadata and finally the handling of semantics for the observed phenomena.

### 3.1. Types of Sensor Discovery

When analyzing which types of sensor discovery have to be supported within the SWE framework, two different search types were identified during the OSIRIS project:

- Sensor instance discovery.
- Sensor service discovery.

Sensor instance discovery means that a user searches for individual sensor instances (or sensor networks). In this case, he is interested in the physical sensing devices. Opposed to this, sensor service discovery provides a more abstract view. The individual sensing device is not within the focus. Instead, the user wants to discover SWE services which offer a certain type of sensor data or provide certain sensor related functionality like alerting or tasking. Although both approaches are fairly different, the distinction between the two discovery types is not absolute. For example, sensor instance discovery can be used as the foundation for enabling sensor service discovery. Sensor instance discovery combined with a mapping between sensor instances and SWE services can enable a basic type of sensor service discovery. Within this paper an approach will be introduced that addresses mainly the needs of sensor instance discovery. Though, as the presented solution manages at the same time also the links between sensors and SWE services the presented solution can also be used for sensor service discovery.

Another important aspect when defining the requirements for sensor discovery deals with the search criteria that have to be supported within search requests. Within the OSIRIS project, the following criteria have been identified as necessary for both types of sensor discovery:

- Thematic: finding sensors or SWE services based on the phenomena (e.g. finding all sensors or services that provide temperature measurements).
- Spatial: finding sensors or SWE services that are related to a certain location or area (e.g. finding all sensors or services that provide data for the city of Münster).
- Temporal: finding sensors or SWE services that offer data for a certain point in time or time period (e.g. finding sensors or services that deliver data measured during the last five days).

Besides these criteria that are common to sensor instance discovery and sensor service discovery, there are also criteria which are only applicable to one of the discovery types. The following two criteria are more related to sensor instance discovery whereas they are not as relevant for the sensor service discovery:

- Sensor properties: finding sensors based on sensor specific (status) properties (e.g. finding all sensors that have a battery level lower than 10%).
- Sensor identification: finding sensors based on their id.

With regard to sensor service discovery the following two criteria often have to be considered:

- Functionality: finding SWE services based on the functionality they are offering (e.g. data access, sensor tasking, alerting).
- Usage restrictions: many SWE services have certain security or usage constraints. They are configured to accept only particular clients with special access privileges (e.g. access only for members of official organizations). Such usage restrictions need to be considered when searching for SWE services.

This list of sensor discovery criteria consists of a common set of queries. However, depending on the domain, additional criteria (e.g. reliability, precision, etc.) may be necessary. Although, the different search criteria have been listed independently within this section, they are usually combined within search requests submitted to a sensor registry or catalogue. For example, a user might search for SWE services that offer temperature data not older than one hour for the city of Münster.

### 3.2. Handling the Dynamic Structure of Sensor Networks

A very important characteristic of sensor networks is their often highly dynamic structure: mobile sensors might move around, new sensors may be deployed, defective sensors may disappear and other sensors may switch between active and sleep mode in order to save energy [17]. In comparison with conventional data sources like static maps or vector based geodata, sensor networks are much more dynamic. This fact has to be considered when building a catalogue or registry for sensors. Especially the following issues have to be addressed within a solution that is adapted to the requirements of dynamic sensor networks:

- Changing availability of sensors and sensor data: Sensors are often only available for certain time periods (i.e. sensors which are powered by batteries have generally a constrained life time). Thus, a sensor registry must handle the metadata about sensors in a time dependent way.
- Keeping track of mobile sensors: In case of moving sensors the locations for which data is available are continuously changing. This has to be reflected by the data model that is used within a catalogue instance (e.g. by relying on the concepts of moving objects databases).
- Quick updates of search indexes: As described before, the metadata of sensor instances and SWE services may change quickly. Besides the use of special data models it is also necessary to create update mechanisms which ensure that any updated meta information is inserted into the data model of the sensor registry as quick as possible.
- Handling changing links between sensor instances and SWE services: Not only the structure of sensor networks may change. Also the links between SWE services and sensors can change during the course of time. Thus, a sensor registry must keep track of the sensor/service associations.

In Section 4.1 the Sensor Instance Registry will be introduced. This registry concept has especially been created in order to address the challenges caused by the often highly dynamic sensor network structure.

### 3.3. Taking into Account Sensor Status Information

Closely related to the dynamic structure of sensor networks is the handling of sensor status information. Although information like the battery state or the health of a sensor is normally not of interest when searching for sensors, it gains a significant importance in the context of sensor network administration. For example, a sensor network operator might want to use a sensor registry for determining which of his sensors need to be serviced (e.g. changing the battery). In this case he needs to be able to submit search requests like “search for all of my sensors located in a certain area that have a battery level below 10%”.

In addition to the use case of sensor network administration there are often situations in the normal sensor discovery process when sensor status information provides a significant improvement. A typical example is the tasking of sensors like controllable cameras. In this case it is obvious that only one user should be able to set the zoom, pan or tilt parameters of such a camera at a certain instant of time. Although the services which provide a tasking interface for such a camera (i.e. SPS) allow feasibility studies for a certain task (i.e. check for availability of the camera), this creates an unnecessary overhead. For user it would be more comfortable to receive the information directly from the sensor registry which sensors are able to fulfill a certain task.

A solution for handling sensor status information will be presented in Section 4.1. There, the Sensor Instance Registry (SIR), which provides an interface for sensor status handling, will be shown.

### 3.4. Describing and Harvesting Sensor Metadata

As sensor networks can comprise up to thousands or even more sensors it is often not feasible to register every sensor manually within a sensor registry. In consequence there is a need for mechanisms that automatically gather required sensor metadata in order to populate the search indexes of a sensor registry with meta information.

For being able to develop such automated solutions, two issues have to be solved:

- How shall sensor metadata be described and encoded in order to interpret it correctly during the insertion into search indexes?
- How can a mechanism for harvesting sensor metadata be designed?

To answer the first question, it is possible to rely on SensorML. It provides a standardized and well-defined sensor metadata format. But for using it as input to automatically extract information, relevant for discovery purposes, SensorML’s data model raises a challenge: The SensorML standard was especially optimized to achieve a maximum of flexibility in order to allow the description of nearly any sensor type that exists. As a result most parts of the SensorML standard possess an optional character. Furthermore, the structure of SensorML documents containing the same information can vary. This makes it extremely difficult to transfer a SensorML description automatically into sensor registry entries. As a solution in Section 4.3 the idea of SensorML profiles will be discussed that allow a more precise definition which information in which structure can be expected by a metadata harvesting mechanism. Additionally, Section 4.3 contains the description of a sensor metadata harvesting solution that allows to automatically analyze SWE services and to extract the according SensorML metadata descriptions.

### 3.5. Dealing with Semantics

For the discovery of spatial data and thus also for geosensor data it is important to consider the semantic issues [18]. In case of sensor networks this becomes especially relevant when describing the phenomena which are observed by the sensors or spatial references [19].

In many cases the observed phenomena are just described by providing a textual identifier. This can lead to a situation in which two strings (e.g. COConcentration and CarbonMonoxideConcentration) refer to exactly the same phenomenon (e.g. carbon monoxide concentration). If a user needs to find sensors which deliver information about a certain phenomenon, he theoretically needs knowledge about all phenomenon names if he wants to receive a complete result. This is at least true in case of a pure text based search mode. However, if the sensor registry is able to recognize that some textual identifiers point to the same phenomenon, a more powerful discovery approach becomes available. In this case the registry would deliver all sensors measuring the same phenomenon, even if it is identified by different phenomenon names.

Another scenario is a situation in which a user does not exactly know, for which phenomena he wants to access sensor data. For example a user requires any kind of weather data. If he sends an according request to a semantically enabled sensor registry, the registry would be able to discover sensors for all phenomena that are somehow weather related (e.g. rain, air temperature, wind speed, etc.).

Besides this phenomenon centric view on semantic also spatial semantics play an important role during the discovery process. This aspect concerns especially managing the links between place names and the places themselves. For example one place name might refer to different locations (e.g. several cities with the same name or a place name that refers to a city as well as a state). In order to enable a more powerful discovery process also spatial semantics are considered. A first solution, the Sensor Observable Registry (SOR), which offers a basic interface for exploiting basic semantic relationships between phenomena observed by sensors, will be presented in Section 4.2.

## 4. The OSIRIS Sensor Web Discovery Framework

In this section a discovery framework for the Sensor Web will be introduced. This framework was specified, implemented and practically tested within the EC funded OSIRIS (http://osiris-fp6.eu/) project. It is integrated within the SWE architecture and enables the discovery of sensor instances as well as sensor services. In Section 4.1 the Sensor Instance Registry (SIR) will be introduced. The SIR is a registry which is capable of managing sensor instances. Thus, the SIR is especially addressing the need of sensor instance discovery. However in the OSIRIS project a need was recognized to manage also the links of sensor instances to sensor services. Thus, also this information is managed within the SIR. As a consequence, the SIR supports a basic approach to sensor service discovery as well. After this in Section 4.2 the Sensor Observable Registry (SOR) for managing the semantics of the observed phenomena will be described. Section 4.3 addresses the issues of metadata formats and metadata harvesting mechanisms required for the SIR to ingest metadata. Finally, in Section 4.4 a potential approach for linking the OSIRIS Sensor Web discovery framework to existing (non sensor specific) catalogues will be discussed. Especially the OGC Catalogue Service is expected to be an important solution for providing complex sensor service discovery functionality in the future.

### 4.1. The Sensor Instance Registry

The concept of the SIR was developed during the OSIRIS project due to the fact that a component was needed which is capable of handling (often highly dynamic) metadata of sensors, discovering sensors and managing the links between sensors and SWE services [20].

Basically, the operations of the SIR can be divided into two sub-interfaces: one for handling sensor metadata and allowing sensor discovery and one for managing sensor status information (i.e. for network administration tasks).

In the sensor discovery part of the SIR interface both, sensor instance discovery and sensor service discovery, are covered. So, a user is able to search for sensors as well as SWE services encapsulating them. In detail the sensor discovery functionality of the SIR interface comprises the following operations:

- SearchSensor: This operation accepts requests that contain a set of user defined search criteria (the phenomenon of interest, spatial and temporal constraints, the unit of measurement, text fragments that shall occur anywhere in a sensor description and IDs of sensors of SWE service instances (e.g. URL of the SWE service)). It returns a list of those sensors which match the search criteria as well as a list for each sensor containing the SWE services which encapsulate the sensor.
- DescribeSensor: In the SearchSensor operation the user receives a list containing the IDs of all sensors that match the search criteria. By submitting a sensor ID in a DescribeSensor request a user is able to retrieve a complete SenorML description for a given sensor.
- HarvestService: This operation can be used in order to analyze a SWE service with a given URL. The operation extracts all metadata provided by the SWE service and inserts it into the SIR search indexes.
- InsertSensorInfo: Sometimes it is not feasible to automatically harvest all necessary metadata from a SWE service (e.g. if the SWE service does not contain a complete metadata set). The InsertSensorInfo operation allows submitting sensor metadata (SensorML descriptions as well as links between sensors and SWE services) manually to the SIR.

In addition to these sensor discovery related operations the SIR does also support the management of sensor status data. Using these operations a user is able to insert any kind of sensor status information, to retrieve information about the status of one or more sensors and to subscribe to certain status information messages based on user defined criteria. This has lead to the following sensor status handling interface of the SIR:

- GetSensorStatus: This operation allows retrieving the status information for a specific sensor but also for a set of sensors fulfilling certain user defined criteria.
- SubscribeSensorStatus: Sometimes a user needs to be informed as soon as certain sensor status events occur (e.g. if a sensor has some kind of error the operator might want to be notified as quickly as possible). The SubscribeSensorStatus operation can be used for submitting such subscriptions to the SIR. (In addition there are also operations for cancelling or extending subscriptions which will not be explicitly described within this article).
- InsertSensorStatus: By calling the InsertSensorStatus operation a sensor operator can submit sensor status information to the SIR. Thus, if a sensor operator uses this method he can ensure that new sensor status data is published as soon as it is available.

Internally, the SIR is currently built on three different types of search indexes: a spatial index for allowing an efficient sensor discovery based on spatial constraints (Within the implementation the Java Spatial Index (JSI) library was used. More information can be found here: http://jsi.sourceforge.net/), a temporal index for handling search requests containing temporal criteria and finally a full-text index which allows searching for keywords that occur within a sensor description (For realizing the full text index the SIR implementation relies on Apache Lucene (http://lucene.apache.org/)). These three indexes are combined by the internal SIR logic. Currently these three indexes are used in parallel. However, for the future more efficient ways for combining these indexes will be investigated.

As the name Sensor Instance Registry indicates, the discovery of sensor instances has been the main goal. In addition, the integration of an internal mapping between the sensors and SWE services has made it possible to serve also basic requests aiming at sensor service discovery. The experiences gained during the practical use of the SIR will be described in Section 5.

### 4.2. The Sensor Observable Registry

The SOR was developed in order to offer an easy approach that allows handling of the phenomena semantics that are observed by sensors in a simple but effective way [20]. On the one hand the SOR can be seen as a dictionary: it manages the definitions of the phenomena and returns on request a textual description as well as a link to a formal definition (i.e. link to an according ontology). On the other hand the SOR is able to exploit basic semantic relationships. It relies on reasoning mechanisms that are applied to the formal definitions of the phenomena in an ontology. Thus, the SOR can determine which phenomena are similar or closely related to another one (e.g. finding specializations or generalizations). The following two operations for the SOR interface have been developed:

- GetDefinition: The GetDefinition operation provides the dictionary functionality of the SOR. This means that it returns for a phenomenon (identified by Uniform Resource Names (URN)) a textual description as well as a reference that links to an ontology in which the phenomenon is formally defined.
- GetMatchingDefinitions: This operation encapsulates the reasoning functionality of the SOR. It accepts the URN of a phenomenon and returns a list of URNs pointing to the definitions of similar phenomena (currently only the specialization-generalisation relationship is exploited, however in the future more complex relationships will be supported).

Internally, the core of the SOR is formed by an XML based dictionary containing an entry for each phenomenon managed by the SOR instance (In the test implementation a phenomenon dictionary is re-used that was developed by participants of the OGC OWS-4 testbed. It uses the dictionary type defined within the Geography Markup Language (GML).). Such a dictionary entry consists of the URN identifying the phenomenon, the textual description and the link pointing to the formal definition in an ontology. In the OSIRIS context the SWEET ontology (http://sweet.jpl.nasa.gov/ontology/) was used. For exploiting the semantic relationships between phenomena the prototypical SOR implementation relied on the Jena Semantic Web Framework (http://jena.sourceforge.net/). Although for the future more complex relationships than those described within the SWEET ontology will be desirable, the results achieved during the OSIRIS project were perceived as a significant step forward compared to a purely text based sensor discovery.

**Figure 2.**
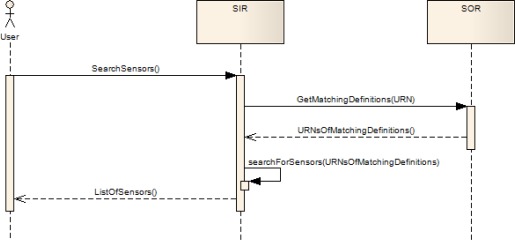
Schema of the interactions between SOR and SIR.

Figure 2 illustrates how the SOR can be used in order to enhance the SIR search functionality. If a user searches sensors that observe a certain phenomenon he sends an according search request to the SIR. The SIR analyzes the search request in order to determine which phenomenon the user is interested in. In the next step the SIR requests a list of definitions from the SOR which match to the one it has received within the search request. The SOR then uses reasoning mechanisms to identify the matching phenomenon definitions and returns a list of URNs of all matching definitions to the SIR. After this the SIR queries textually its search indexes for all sensors which observe one of the phenomena identified by one of the URNs contained in the response received from the SOR. In a last step the SIR returns a list of discovered sensors to the user.

As a result the SOR allows for example the handling of following situations:

- The URNs “urn:standardisationOrganisationA:carbonMonoxideConcentrations” and “urn:standardizationOrganisationB:COConcentrations” might refer to the same phenomenon defined in the same ontology: the concentration of carbon monoxide. The SIR can then find out by requesting the SOR that both URNs possess the same meaning.
- In a crisis situation (e.g. a chemical accident) the firemen need to know which chemicals have been emitted. Thus, they need to find all sensors that measure any kind of air pollutant concentration. Within an ontology it can be modeled that the phenomena identified by the URNs “urn:standardisationOrganisationB:COConcentrations” and “urn:standardisationOrganisationB: NO2Concentrations” are both air pollutant concentrations. Thus, if the SIR receives a request for sensors measuring “urn:standardisationOrganisationB:AirPollutantConcentrations” it can request the SIR for phenomena that are some kind of air pollutant concentration. In this case the SOR would return the according URNs of the matching phenomenon definitions and the SIR could query its search indexes for these URNs.

These two examples show two relatively simple but common scenarios that can benefit from the SOR concept. However, in future the exploitation of more complex semantic relationships seems to be promising as well as the use of sophisticated measures for the similarity between two concepts.

### 4.3. Metadata Formats and Harvesting

In Section 4.1 the SIR, a powerful service for sensor discovery, has been described. However, for effectively using the SIR it is necessary to insert the relevant metadata about sensors and Sensor Web services into the search indexes. This requires on the one hand a common metadata format that can be interpreted by the SIR and on the other hand a harvesting mechanism that automatically analyses Sensor Web services and the encapsulated sensors so that the according metadata are fed into the SIR. These two aspects will be addressed within this section.

Section 2.3 introduced SensorML as the data format within the SWE framework which covers the description of sensor and sensor system metadata. A SWE discovery solution should consequently use SensorML as its input format for registries and catalogues. However, a SWE discovery approach has to cope with the high flexibility of the SensorML data model. The SensorML data model specifies a majority of its elements as optional. Further on, SensorML allows expressing the same information in several, differently structured ways. This open and flexible structure was one of the main aims of the SensorML design in order to make it possible to apply the data model to nearly any type of sensor. For ensuring that SensorML documents which are intended for discovery purposes can be reliably handled by an automatic harvesting mechanism, it is necessary to create a profile for SensorML that defines the information which must be contained in a SensorML document as well as the structure in which the metadata must be encoded.

Within the OSIRIS project a first version of such a SensorML profile was developed. Based on the requirements of the project the following information items were identified as necessary [20]:

- Names and/or identification of the sensor (id within a SWE service and if possible also service independent id).
- Keywords providing important terms that describe the sensor.
- Information about the company/individual that operates the sensor.
- Classification of the sensor (e.g. sensor type).
- Location of the sensor and/or the observed area.
- Phenomena that are observed by the sensor.
- Outputs of the sensor (e.g. data types and unit of measurement).
- A description of the time spans for which the SensorML document is valid (this is especially important in case of dynamic sensor networks like those consisting of mobile sensors).

In the context of the OSIRIS project these information items form the basis for defining a SensorML discovery profile. This profile was defined in a formal way by using the Schematron XML schema Language [21]. A Schematron schema then serves as a separate extension of the existing SensorML schema. Within this schema extension certain optional elements of the original schema can be redefined as mandatory. In consequence, SensorML documents can be validated against the Schematron schema to fit the special needs of sensor discovery. The so defined profile ensures that all necessary metadata is provided in the correct structure. This validation against the Schematron schema can especially be used by sensor operators/providers in order to create correct descriptions of their sensors. For example editors for SensorML documents might use the profile in order to allow users to check the correctness of their sensor descriptions.

Currently the OSIRIS SensorML discovery profile describes the minimum requirements that have to be fulfilled in order to deliver complete sensor metadata to a SIR instance. During the definition of the profile strong emphasis was put on requiring only the absolute minimum of metadata as there is a risk to require information that is not applicable in every case. However a need for discussion involving a broad range of actors remains in order to ensure the portability between different domains and scenarios.

The current version of the SensorML discovery profile provides an early basis for a discussion on the definition of such a profile. As the OSIRIS results were also used as an input for the OGC OWS-6 testbed, an updated version of this profile will be published separately as an OGC engineering report. In this report the SensorML profile will be described in details and a practical example of a resulting SensorML document will be provided. This might stimulate further discussions within the OGC community regarding the definition of such a profile. The engineering report will contain the formal specification of the SensorML profile as well as a set of examples. It is planned to publish this engineering report as an OGC discussion paper after the results have been presented at an OGC Technical Committee meeting in summer 2009.

As it can be expected that in other contexts additional metadata items may become necessary, there might arise a need for further domain-specific SensorML profiles. However in order to ensure consistent approaches for the definition of such profiles, for the future some guidelines and rules for the definition of SensorML profiles might be interesting.

As the formats for describing required sensor metadata are now defined, a next step describes an approach which is capable of automatically harvesting the according metadata documents.

The harvesting mechanisms for retrieving metadata are making direct use of the according operations as specified within the different OGC standards. In order to avoid redundancies, this article focuses on the SOS and the SPS Service whereas the SAS is not explicitly described as the harvesting works similar to the SPS. The WNS will also not be addressed as it is a helper service within the SWE framework which provides no directly sensor related functionality.

Like all OGC web services the SOS offers the GetCapabilities operation. This method returns a general description of the according service instance including information about available operations, provided data and the service provider. In case of a SOS this is general information about the content of the service including a list of sensors. Within this sensor list an id for each sensor is given which can subsequently be used for requesting more detailed metadata. This is achieved through the DescribeSensor operation which returns the according SensorML document for each sensor (defined by a parameter containing the sensor id).

Due to the fact that not every SWE service offers the DescribeSensor operation (currently it is limited to the SOS) a further mechanism that covers services like the SPS is needed. Like in the SOS example, at first a Capabilities document of the SPS instance is retrieved. However, within this document there is no reference to sensor ids which could be re-used in DescribeSensor requests, as this operation is not defined for the SPS. Instead the Capabilities document can offer direct links to the according locations of the necessary SensorML documents.

**Figure 3.**
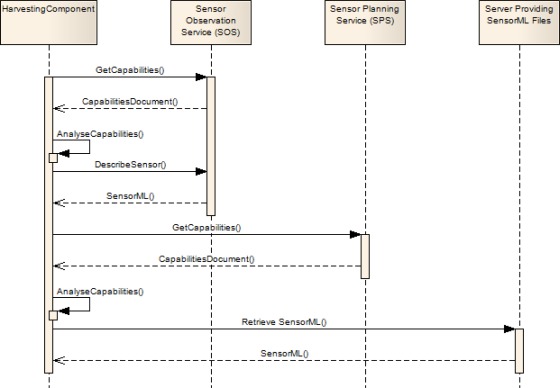
Schema showing of the SIR metadata harvesting mechanism.

Figure 3 shows a schematic illustration of the sensor metadata harvesting mechanisms described above.

After all SensorML documents have been retrieved, the SIR is able to analyze their content and to integrate the harvested metadata into spatial, temporal and thematic indices which enable the search according to discovery requests the SIR receives.

It is clear that not all SensorML documents comply with the SensorML discovery profile described before. Currently such SensorML documents are not inserted into the SIR. However for the future it will be investigated how at least a certain amount of robustness for handling not fully compliant SensorML documents can be achieved.

### 4.4. Linking the OSIRIS Sensor Discovery Framework to the OGC Catalogue

In the area of spatial data infrastructures the topic of catalogues is already covered by the OGC Catalogue Service (CS-W). Within spatial data infrastructures the CS-W allows searching for geospatial data as well as geospatial web services. As the CS-W has already found a broad acceptance and use in practical applications, it can be seen as the de-facto discovery solution that must be offered when publishing any kind of data or service within a spatial data infrastructure. Consequently, the link between the SWE discovery framework and the CS-W is of great importance.

The SIR presented in section 4.1 is to a high degree specialized to take into account the specific properties of sensor networks. By providing mechanisms for including characteristics like the dynamics of sensor networks or the exploitation of sensor status information, it goes beyond the scope of the CS-W. This creates the need for investigating how the specialized sensor discovery functionality of the SIR can be mapped to existing CS-W instances. As a result there will be a two-tiered approach: The CS-W for searching SWE resources within spatial data infrastructures on a more general level and the SIR comprising discovery mechanisms closely adapted to the needs of sensor networks.

The work on achieving this link is still in progress. A pragmatic first step will be to map the SWE metadata formats to the CS-W data models. At the moment efforts are made by several groups in order to create a mapping between SensorML and two of the most important catalogue data models: ebRIM and the standardized ISO 19115 meta data model (At least the following activities which deal among other topics with a CS-W for sensors and/or SWE service are known to the authors: The EC funded projects SANY (http://sany-ip.eu/) and GENESIS (http://genesis-fp7.eu/) and the OGC OWS-6 testbed. However, the authors are not aware of publically available detailed documents of these projects that could be referenced.). This mapping will make it possible to insert sensor metadata into a catalogue and to use such a catalogue for searching sensors. However, this is a more general approach that does not specifically address issues like the dynamics of sensor networks or continuously changing sensor status properties. Furthermore, a mapping between sensors and SWE services is not achieved by just translating SensorML to a catalogue data model.

In a next step the mapping between SensorML and the different catalogue data models can be exploited to develop a more sophisticated metadata feeding concept. A first idea is based on the already mentioned two-tiered approach. The SIR remains responsible for sensor discovery on a lower level that is closer to the sensor networks themselves. On this level the SIR keeps its powerful interface. Furthermore the SIR can be enhanced by a feeding mechanism that transfers sensor metadata to a conventional catalogue. An important task of the SIR with regard to this feeding mechanism will be to aggregate and generalize the metadata transferred to the CS-W in order to reduce the amount of data and the update frequency the CS-W has to deal with. For example in case of a continuously moving sensor, it is normally sufficient to transmit the updated position only in certain time intervals and to abstract the area the sensor is moving in by describing a geometry like a bounding box. As a result, users who need to access the sophisticated SIR functionality can use the SIR interface whereas other users who are just searching for example sensor data can access a CS-W instance. In the latter case the mechanisms for searching sensors or sensor data would be the same approach like the discovery of any other resource within a spatial data infrastructure.

## 5. Experimental Evaluation

The SWE discovery framework presented before (except the link to the OGC Catalogue Service) has been implemented during the OSIRIS project. Furthermore the SIR implementation has been published as free software through the open source initiative 52° North (further information and documentation about this implementation can be found on the web site of 52° North: http://52north.org/index.php?option=com_content&view=article&id=299&Itemid=130).

Within the OSIRIS project, in cooperation with 12 other European partners, four different scenarios have been used as practical background for testing all OSIRIS SWE implementations and enhancements. In detail the following four use cases were addressed [22]:

- Forest fire fighting: In this scenario aerial image data as well as real-time positioning systems have been used for achieving an improved early fire detection, a more accurate situation assessment as well as efficient means for planning the fire fighting resources.
- Air pollution: This scenario was realized in the city of Valladolid (Spain). Mobile sensors (mounted on buses), stationary sensors as well as an unmanned airplane were used for collecting air quality data. As a result two sub-systems were completed that allow on the one hand the continuous air quality monitoring and on the other hand the management of accidentally caused air pollution.
- Fire detection in industrial buildings: For realizing this scenario several types of sensors (smoke, temperature, cameras) have been used to built a more reliable fire detection system. A special focus was put on the intelligent combination of sensors in order to avoid false alarms.
- Water pollution: In the Tuscany region in Italy a system was deployed for monitoring the ground water quality with regard to a natural Arsenic concentration as well as accidental spills of hydrocarbons.

As these four scenarios show, the background of the different sensor applications and the sensor types were quite heterogeneous. Thus, these four scenarios were well suited to test the SWE discovery solution. Within the OSIRIS project one SIR instance was deployed that was capable of managing all sensors used within the project.

In order to illustrate the test scenarios of the OSIRIS project a bit more, one of the scenarios shall be presented more detailed: the air pollution scenario deployed in the city of Valladolid (Spain). This scenario is selected as it comprises a representative selection of sensors used within the whole OSIRIS project. There are two main goals that had two be fulfilled by the OSIRIS system in this scenario:

- Continuous air quality monitoring: For fulfilling this aim, two types of sensors were deployed. Besides a mobile sensor (mounted on a bus of the local public transport company) a set of 9 fixed air quality stations was used. The data of these sensors was made available through SOS. Based on these SOS instances a central data processing centre was able to access the data for calculating maps that show the real-time air pollution situation.
- Crisis management in case of accidentally caused air pollution: In this scenario the aim was to predict how an air pollutant cloud develops after an accident. Besides the sensors used in the monitoring scenario (see above) several additional sensors were deployed: a weather station delivering meteorological data, on-demand deployable air quality sensors and an unmanned air plane for exploring the pollutant distribution with a pollutant cloud three dimensionally. Also in this case the resulting sensor data were made available through SOS instances. A simulation model that produced maps showing the degree of danger for the population in the area around the accident site was subsequently able to access the sensor data.

As this description shows, the sensors deployed in the scenario were quite heterogeneous. There were conventional stationary sensors like the weather stations, a mobile bus-mounted sensor, ad-hoc deployable air quality sensors and even a sensor moving in the three dimensional space. Within the scenario implementation, the OSIRIS sensor registry solution was especially used for keeping track of the available sensors and for gaining an overview of the sensor availability. Thus, on top of the sensor registry a sensor network management application as well as a web based display showing the available sensors were built.

The main acceptance criterion during the tests performed within the OSIRIS project was the question if the requested functionality is fulfilled. Especially the following questions were tested during the practical experiments:

- Is the sensor metadata harvesting mechanism reliable?
- Is the sensor registry capable of handling the addition or removal of sensors?
- Is the sensor instance registry able to handle continuous sensor status updates? A special focus on a scenario in which the positions of firemen during a forest fire were tracked. Here update rates of more than one per second occurred.
- Are the users of the sensor registry satisfied with the functionality or is any functionality missing?
- Do the semantic extensions provided by the SOR provide benefit to the users?

The experiments conducted within the OSIRIS project proved the practical usability of the presented discovery framework. Especially the following experiences shall be mentioned:

- Regarding the harvesting mechanisms the experiments showed that they work in a reliable manner. However, this reliability resulted mainly from a common agreement between the OSIRIS partners to provide the sensor metadata using the common SensorML discovery profile. As this agreement does not exist outside the project, there will be a need for developing on the one hand more robust harvesting mechanisms that do not require the compliancy to this profile. On the other hand it is planned to bring this profile to a broader community and into the OGC discussion process in order to integrate other view points. Furthermore the integration of optional discovery relevant metadata is planned within a next step.
- The addition or removal of sensors through the according update operation of the sensor registry worked without any problems. Thus, the dynamicity created by the deployment or removal of sensors can be handled. Although no exact numbers were measured, the registry was able to handle several addition or removal requests per second.
- The handling of sensor status updates worked well. Also in scenarios with often occurring status updates (e.g. changing bus positions or positions of moving firemen) no problems occurred. Update rates of up to 1 per second were tested. More tests with bigger numbers of sensors and even higher update frequencies will be conducted in the near future by using a simulation framework that is currently under development.
- The functionality of the sensor registry was sufficient for the users involved in the OSIRIS project. The next step will be to validate the spectrum of functionality against the needs of further users in different domains.
- The basic capabilities of the SOR for handling the semantics of the observed phenomena have been perceived as very useful. However, for the future more sophisticated tools for expressing the relationships between phenomena are desirable. Especially the integration of means for expressing the similarity between phenomena could provide interesting additional capabilities.
- Due to the limited number of parties that were involved in the project it was easy to agree on a common dictionary for the phenomena that are observed. In practice the development of a phenomenon dictionary will be more important as well as making use of semantic annotations.
- Finally, the link between the developed SWE discovery framework and the OGC catalogue has not been implemented within the OSIRIS project. This will be a task of future projects.
- The performance within the OSIRIS test scenarios was satisfying.

As a summary it can be stated that the evaluation of the SWE discovery framework was successful and that it provides a valuable basis for future developments.

## 6. Conclusions

This article has introduced a framework for the web based discovery of sensors and sensor services. Besides a sensor registry which is capable of handling the dynamic properties of sensors as well as related metadata formats and harvesting mechanisms, it provides functionality to handle semantics of observed phenomena. The framework as it is described within this article has been successfully tested in the OSIRIS project where it was used in applications ranging from fire fighting to monitoring different kinds of pollution.

However, for the future several challenges remain. Currently, the presented framework is in a prototypical state and needs to be advanced to a stable architecture. In this context it will also be important to further align the framework with the OGC activities and to bring it into the OGC discussion process. Furthermore, the handling of semantics in the context of sensor discovery and the link to existing catalogues need to be addressed more extensively in the future.

However, already in its current prototypical state the presented discovery framework has been proven to be an effective solution within the SWE framework. It will be an important basis for further developments and approaches in the context of Sensor Web discovery.
